# Anesthesia and global warming: the real hazards of theoretic science

**DOI:** 10.1186/2045-9912-2-7

**Published:** 2012-03-25

**Authors:** George Mychaskiw II

**Affiliations:** 1 DO, FAAP, FACOP, Orlando, FL 2012, USA

## Abstract

Recent speculative articles in the medical literature have indicted certain inhalational anesthetics as contributing to global warming. This unfounded speculation may have deleterious patient impact

## 

"There are three kinds of lies: lies, damned lies and statistics".

-Leonard H. Courtney

Throughout most of the world, surgical anesthesia is provided by the use of various gasses or vapors. In the late 1800's, this was accomplished by ether, but has now been refined to be through the action of (usually) one of three different pharmaceutical products. These products are liquids that emit a vapor which, when breathed by a patient, produces a state of general anesthesia. The vapor is exhaled essentially unchanged and then vented to the outside atmosphere, usually through the suction system of hospitals. The drugs are commonly termed inhalational anesthetics. Each product has certain distinguishing characteristics that make it more or less appropriate for use in a particular patient, but they all share the qualities of stability and non-flammability. They are all organic compounds containing halogen atoms, such as chlorine and fluorine. All of the inhalational anesthetics absorb infrared light and this property is useful to anesthesia practitioners in their measurement. In the most common way of measuring the concentration of an inhalational anesthetic that a patient is breathing, a beam of infrared light is passed through the gas as it flows from an anesthesia machine. The anesthetic vapor absorbs a certain amount of infrared light and, by measuring the amount absorbed, a concentration can be calculated. Until recently, any environmental consequence of liberation of the inhalational anesthetics was not given much thought, considering their infinitesimally small proportion of the overall atmosphere. That all changed, however, in 2010.

In 2010, Dr. Susan Ryan, from the University of California, San Francisco, published an article in the journal Anesthesia and Analgesia, which calculated the potential environmental impact of inhaled anesthetics in the atmosphere, based on extrapolation of their absorbance of infrared light. That is, if an anesthetic absorbs X amount of infrared light and is used Y times daily in Z number of operating rooms, then multiplying everything together can result in a certain amount of global warming potential [1]. Sounds complicated? It is. Basically, Dr. Ryan took a mathematical theory and concluded that the environmental impact of using a certain drug was the equivalent of driving 200 odd miles, while using another was the same as only driving 30 miles, etc. So what the authors were doing was speculating on potential climate impact if one believed all of their mathematical assumptions and yet, as I'll explain below, chose to ignore others that seem equally well supported. Of course, in the world of carbon offsets and green technology, this makes for good reading and the article was accompanied by press releases announcing the impact of anesthesia on environmental health and was followed by media hype and further articles about the environmentally ethical selection of certain anesthetics [2]. The Ryan and subsequent articles, took particular umbrage at the use of nitrous oxide, also called "laughing gas" and desflurane, one of the inhalational anesthetics, as it had a greater theoretical global warming potential. Since nitrous oxide is falling out of favor in anesthesia and because desflurane was, until very recently, a proprietary product of a large pharmaceutical firm, it became the subject of many popular media attacks, including one calling for its ban altogether.

All of this might make for an amusing bumper sticker to put on your Prius, were it not for the very real patients that are being placed in jeopardy because of this mathematical extrapolation. Desflurane has some particularly desirable characteristics, including a very low solubility in the tissues of the body that allows patients to wake up faster after it is discontinued [3]. This is an especially important consideration when giving anesthesia to patients who, like many Americans, are overweight or obese. Although there are many different ways to administer anesthesia, most practitioners agree that desflurane offers important safety advantages when caring for the obese and overweight and to have these individuals awake quickly is a good thing and, by extension, a quick wake-up is probably valuable for all patients [4]. So, it was with some dismay that I learned a certain well-regarded Ivy League university hospital had substantially decreased its usual use of desflurane in a misguided quest to be "environmentally ethical" and there were even calls among certain faculty members for the drug to be banned entirely from the hospital's supply (personal communication, J. Sherman MD). Of course, the counter argument is that there are alternative inhaled anesthetics that are nearly as good and have a lower global warming potential, that is, only driving 30 miles in our hypothetical car instead of 200.

Since we have crossed into the world of funny statistics and assumptions, let's look at this issue in terms that are a little more relevant, or at least understandable. Let's assume that the amount of CO2 produced globally every year (50 billion tons) [5] is equivalent to a two-dimensional area the size of the State of Kansas (82,282 sq mi) [6]. Taking into account the amount of CO2 equivalent accounted for by CFC's (2.5 billion tons) [7], the proportion of inhaled anesthetics that make up CFC production (.014%) and the approximate global market share of desflurane (10%) [8], the equivalent area of global warming potential for desflurane is about 300 square feet, or a small apartment in San Francisco. So, because of 300 square feet in an area the size of Kansas, otherwise rational professionals, who normally practice evidence-based medicine, choose to use a drug that, although offering definite patient care advantages, is not as "environmentally ethical" as some of the others. Don't like that example? We could use the Empire State Building. If all the CO2 produced globally each year is represented by the Empire State Building, then the amount contributed to by desflurane is, theoretically, a building about 0.3 mm tall (watch your head). I could go on and on. Now, one of the other erroneous assumptions that Ryan and company make is that this desflurane is sticking around forever, so one 300 square foot apartment is piled atop another, ad infinitum, until the thermostat starts rising. Again, this is not true. Even under worst case assumptions, desflurane has a theoretic life in the atmosphere of around 20 to 30 years, versus CO2's 100 [9]. It should also be noted that, as it contains neither chlorine nor bromine, desflurane does not react with or contribute to ozone depletion [9], but again, that is one of the facts best ignored when drumming up an indictment. You may wonder why I continue to use terms like theoretical, maybe and possibly? Because these are all mathematical projections, like my projection of the 300 square foot apartment. The fact is, none of the inhaled anesthetics has ever been detected in the upper atmosphere. None, zip, zero, at least in any amount that contemporary technology can measure.

Much scientific literature is complex, arcane and generally irrelevant, except that it serves as a rung in the academic climb of the ladder from assistant to associate professor, and so forth. Generally these small papers are of little consequence and impact the community only by the felling of trees for dusty medical journals. In certain circumstances, however, such as the perfect storm of climate awareness, big pharma bashing and environmental "ethics", misinterpretation of this literature can hurt people in the real world. As one of many Americans who is overweight, I would only ask my future anesthesia practitioners to do what they do best: administer the anesthetic that is safest for me, and leave the speculation about global warming potential to the environmental engineers.
